# Oxysterol binding protein-related protein 8 mediates the cytotoxicity of 25-hydroxycholesterol[S]

**DOI:** 10.1194/jlr.M069906

**Published:** 2016-10

**Authors:** Jiwei Li, Xiuting Zheng, Ning Lou, Wenbin Zhong, Daoguang Yan

**Affiliations:** Department of Biotechnology* Jinan University, Guangzhou 510632, China; Key Laboratory of Functional Protein Research of Guangdong Higher Education Institutes,† Jinan University, Guangzhou 510632, China; State Key Laboratory of Oncology in Southern China,§ Collaborative Innovation Center of Cancer Medicine, Guangzhou 510060, China

**Keywords:** cytotoxicity of oxysterols, endoplasmic reticulum stress, apoptosis

## Abstract

Oxysterols are 27-carbon oxidized derivatives of cholesterol or by-products of cholesterol biosynthesis that can induce cell apoptosis in addition to a number of other bioactions. However, the mechanisms underlying this cytotoxicity are not completely understood. ORP8 is a member of the oxysterol binding protein-related protein (ORP) family, implicated in cellular lipid homeostasis, migration, and organization of the microtubule cytoskeleton. Here, we report that 25-hydroxycholesterol (OHC) induced apoptosis of the hepatoma cell lines, HepG2 and Huh7, via the endoplasmic reticulum (ER) stress response pathway, and ORP8 overexpression resulted in a similar cell response as 25-OHC, indicating a putative functional relationship between oxysterol cytotoxicity and ORP8. Further experiments demonstrated that ORP8 overexpression significantly enhanced the 25-OHC effect on ER stress and apoptosis in HepG2 cells. A truncated ORP8 construct lacking the ligand-binding domain or a closely related protein, ORP5, was devoid of this activity, evidencing for specificity of the observed effects. Importantly, ORP8 knockdown markedly dampened such responses to 25-OHC. Taken together, the present study suggests that ORP8 may mediate the cytotoxicity of 25-OHC.

Oxysterols are 27-carbon oxidized derivatives of cholesterol or by-products of cholesterol biosynthesis. These compounds act as intermediates of cholesterol catabolism and, when accumulating in pathologic situations, display cytotoxic and pro-apoptotic activities (1–4). Importantly, research in the past decades has revealed additional physiological activities of the endogenous cellular oxysterols: they act as signaling molecules with regulatory impacts on diverse aspects of cell biology, including lipid metabolism, signal transduction, immune function, developmental processes, cell cycle regulation, and apoptosis (5–7). Of the oxysterols, 25-hydroxycholesterol (OHC) is the most extensively studied and has been implicated, among other functions, in cell death (8, 9).

Disruption of the normal function of the endoplasmic reticulum (ER) causes a stress response known as the unfolded protein response, initially aimed at compensating for the damage (10, 11). ER stress results in the abnormal accumulation of unfolded and misfolded proteins due to the limited protein-folding capacity of the ER (12, 13). If the defensive unfolded protein response fails to deal with the misfolded proteins in the ER, ER stress-induced apoptotic death signaling is activated (14). Oxysterols are derived from cholesterol oxidation and are found in oxidized LDLs that act as lipotoxic agents inducing ER stress (15). Also, 25-OHC has been reported to induce ER stress and apoptosis in macrophages (16).

Oxysterol binding protein was isolated in the 1980s as a cytoplasmic high-affinity receptor for several oxysterols (17–19). Oxysterol binding protein-related proteins (ORPs) comprise a 12-member gene family in mammals (20). ORP8 is a member of the ORP family that contains a single C-terminal transmembrane domain targeting the protein to the ER. Our previous study indicated that ORP8 decreases cholesterol efflux in macrophages by suppressing ABCA1 expression, implying that it may play a role in the development of atherosclerotic lesions (21). In hepatic cells, ORP8 functions as a negative regulator of intracellular cholesterol (22). Other roles of ORP8 have been suggested, including the inhibition of cell migration through interaction with nucleoporin Nup62 (23), mediation of oxysterol interference of the cell cycle through interaction with astrin/SPAG5 in HepG2 cells (24), and transport of phosphatidylserine at ER-plasma membrane contact sites (25). Importantly, OPR8 increases the sensitivity of hepatocellular carcinoma (HCC) cells to Fas-mediated apoptosis (26). There is evidence that ORP8 acts as an oxysterol binding protein (21, 27), indicating a possible mechanistic link between 25-OHC and ORP8 in cell apoptosis.

In the present study, we provide evidence that 25-OHC induces ER stress and cell apoptosis in HepG2 and Huh7 cells, and that ORP8 may mediate these cellular responses.

## MATERIALS AND METHODS

### Materials

The 25-OHC, 7-OHC, 4-PBA, and Hoechst 33342 were from Sigma-Aldrich. The 27-OHC and 7-ketocholesterol (7-keto) were from Santa Cruz. The FITC annexin V apoptosis detection kit I was from BD Biosciences. Cell Counting Kit-8 (CCK-8) was from Dojindo.

### cDNA constructs and transfection

Human *ORP8* cDNA (NM_001003712) and truncated *ORP8* cDNA [ORP8 without the C-terminal ligand-binding ORD domain (designated ORP8△ORD)] were inserted into the *Bgl*II and *Sal*I sites of pcDNA4HisMaxC (Invitrogen, Carlsbad, CA) to obtain constructs expressing proteins fused with an N-terminal Xpress epitope tag. *ORP5* cDNA (NM_020896) was inserted into the *Xba*I site of pcDNA4HisMaxC. The primers used are listed in supplemental Table S1. Transient transfections of cultured cells were carried out using Lipofectamine 2000 (Invitrogen) according to the manufacturer’s instructions.

### Cell culture

HepG2 cells and Huh7 cells were cultured in DMEM supplemented with 10% FBS (pH 7.4), penicillin (100 U/ml), and streptomycin (100 μg/ml). Cells were maintained in 5% CO_2_, 37°C.

### RNA interference

One day before transfection, HepG2 or Huh7 cells were seeded on 12-well plates at 30–50% confluency and then transfected with siORP8, siORP5, or control nontargeting siRNA (siNT) for 72 h (siORP8, GAGUGGUCUUGCAAAUUAUdTdT; siORP5, CCCUGCCCAGCAGCUACCUGAUCdTdT; and siNT, UAGCGACUAAACACAUCAAdTdT) by using Lipofectamine 2000 (Invitrogen).

### Quantitative real-time PCR

Total RNA was isolated with TRIzol reagent (Invitrogen; according to the manufacturer’s instructions) from HepG2 or Huh7 cells. RNA samples were reverse transcribed using random hexamer primers in the presence of RNase inhibitor (Takara Bio, Shiga, Japan). Quantitative (q)RT-PCR was performed with SYBR Premix EX Taq (Takara Bio) using the 7300 sequence detection system (Life Technologies/Applied Biosystems, Carlsbad, CA). The primers used are shown in supplemental Table S1. Relative quantification analysis was performed using the ΔΔCt method with actin as endogenous control; relative gene expression was presented as the ratio of the target gene to reference control.

### Cell proliferation assay by CCK-8

Cells were plated in 96-well plates. After a 24 h culture, cell numbers were evaluated by CCK-8 following the manufacturer’s protocol. Cell number was calculated by the standard curve method, and the averages of at least three independent experiments are presented.

### Hoechst 33342 staining analysis

Hoechst 33342 staining was performed to observe the nuclear morphological changes in HepG2 or Huh7 cells. The cells were collected, washed twice with PBS, and then incubated with Hoechst 33342 (10 μg/ml) for 10 min. Then, the cells were washed with PBS and observed by fluorescence microscopy using appropriate filters for blue fluorescence.

### Annexin V-FITC/propidium iodide staining for apoptotic stages

Flow cytometry was performed on the 25-OHC-treated HepG2 cells to observe the effects of 25-OHC on cell apoptosis with FITC annexin V apoptosis detection kit I. The 25-OHC-treated cells were cultured, harvested at the indicated times, washed twice with cold PBS, and then resuspended in 1× binding buffer at a concentration of 1 × 10^6^ cells/ml. One hundred microliters of the solution (1 × 10^5^ cells) were transferred to a 5 ml culture tube, 5 μl of FITC annexin V and 5 μl propidium iodide (PI) were added, gently vortexed, and the cells were incubated for 15 min at room temperature (25°C) in the dark. Then 400 μl of 1× binding buffer was added to each tube, followed by analysis by flow cytometry within 1 h.

### Western blot analysis

Cells treated under different conditions were washed twice with ice-cold PBS, scraped from the dishes, and suspended in lysis buffer [50 mM Tris-Cl (pH 8.0), 150 mM NaCl, 0.5 mM MgCl_2_, 10% glycerol, 1% Triton X-100, 0.1% SDS] with protease inhibitor cocktail (Roche Diagnostics, Basel, Switzerland) on ice for 10 min before clearing of the lysates by centrifugation for 5 min at 12,000 *g*. Lysates were separated by SDS-PAGE and transferred to PVDF membranes. For detection of the proteins, the membranes were incubated with anti-phospho-protein kinase RNA-like ER kinase (PERK) (catalog number sc-32577, Santa Cruz), anti-PERK (catalog number sc-13073, Santa Cruz), anti-phospho-eukaryotic translation initiation factor-2α (eIF2α) (catalog number sc-101670, Santa Cruz), anti-eIF2α (catalog number sc-11386, Santa Cruz), anti-CCAAT/enhancer-binding protein-homologous protein (Chop) (catalog number #895, Cell Signaling), anti-activating transcription factor-4 (ATF4) (catalog number 11815, Cell Signaling), and anti-β-actin (catalog number 3700, Cell Signaling), anti-Xpress^TM^ (catalog number R910-25, Invitrogen), anti-ORP5 (catalog number ab127171, Abcam), anti-ORP8 (produced and affinity-purified in our laboratory) respectively, and then incubated with HRP-conjugated anti-mouse IgG (catalog number 7076, Cell Signaling) or HRP-conjugated anti-rabbit IgG (catalog number 7074, Cell Signaling). Protein concentrations of the lysates were measured by the Bio-Rad (Hercules, CA) Dc assay.

### Statistical analysis

Experimental results were analyzed by using the SPSS.21 software. The data are presented as mean ± SD. All comparisons between groups were made by unpaired two-tailed Student’s *t*-test. *P* < 0.05 was considered statistically significant.

## RESULTS

### Oxysterols enhance cell proliferation and apoptosis in a dose-dependent manner

**Fig. 1.**
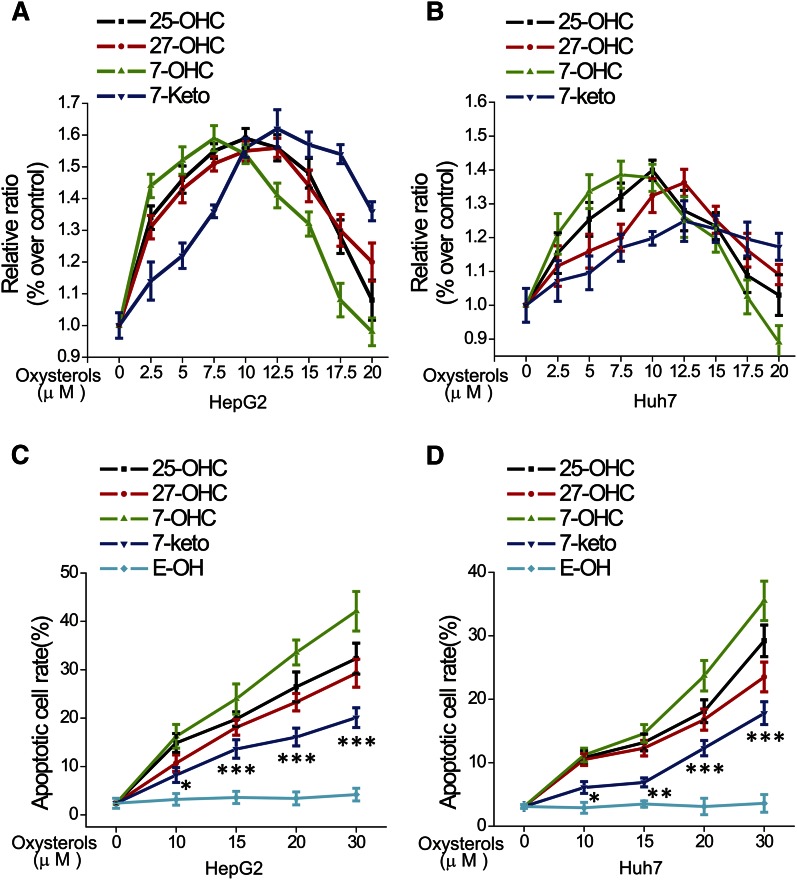
Oxysterols induce proliferation and apoptosis in HepG2 and Huh7 cell lines. A, B: HepG2 and Huh7 cells were incubated for 24 h in the presence of different concentrations of 7-keto, 7-OHC, 25-OHC, and 27-OHC, and then the proliferation rate was detected using CCK-8. C, D: HepG2 and Huh7 cells were treated with different concentrations of 7-keto, 7-OHC, 25-OHC, 27-OHC, and ethanol for 24 h, and then the nuclear morphology was observed under a microscope after Hoechst 33342 staining. The data represent mean ± SD from three individual experiments (n = 3, *P < 0.05, **P < 0.01, ***P < 0.001).

Oxysterols have potent effects on cell growth and death, including induction of apoptosis (5, 28). To assess the cytotoxicity of oxysterols, the effects of 7-keto, 7-OHC, 25-OHC, and 27-OHC on the proliferation of HepG2 and Huh7 cells were measured using CCK-8. As shown in **Fig. 1A, B**, concentrations below 10 μM of 7-keto, 7-OHC, 25-OHC, and 27-OHC promoted cell proliferation, while at concentrations above 10 μM, oxysterols caused a reduction of cell numbers. To further analyze the cytotoxicity of the oxysterols, we employed nuclear staining with Hoechst 33342 in HepG2 and Huh7 cells to determine the proportion of apoptotic cells in the 7-keto-, 7-OHC-, 25-OHC-, and 27-OHC-treated specimens. Compared with the control, the results showed that the number of apoptotic cells increased in a dose-dependent manner upon incubation with all oxysterols (Fig. 1C, D). Of note, HepG2 cells and 10 μM 25-OHC were selected for most of the experiments in this study. Even though this concentration induced stimulation of cell proliferation, it also had a pronounced pro-apoptotic effect (apoptosis rate 14.9% vs. 2.4% in the control). If ORP8 overexpression (see the results below) was combined with 25-OHC concentrations >10 μM, excessive cell death was induced, making it difficult to precisely analyze the apoptotic cell rate (supplemental Fig. S1).

### 25-OHC induces ER stress and cell apoptosis

**Fig. 2.**
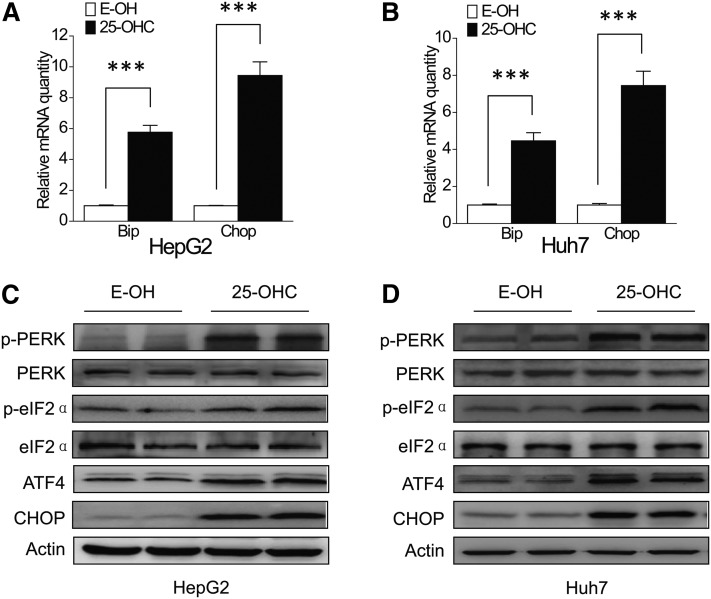
The 25-OHC induces ER stress in HepG2 and Huh7 cells. A, B: HepG2 and Huh7 cells were treated with 10 μM 25-OHC for 24 h, and relative Bip and Chop mRNA levels were measured by qRT-PCR. C, D: The expression of ER stress markers, ATF4, Chop, phospho-PERK, and phospho-eIF2α, were determined by Western blotting. β-Actin was used as a loading control. The data represent mean ± SD from three individual experiments (n = 3, ***P < 0.001).

A previous report showed that 25-OHC could induce ER stress and apoptosis in macrophages (16). To determine whether ER stress was induced by 25-OHC in HepG2 and Huh7 cells, we first examined the expression of immunoglobulin heavy chain-binding protein (Bip) and Chop mRNAs, central components involved in ER stress responses (29). qRT-PCR analyses revealed that the Bip and Chop mRNAs were robustly induced after 24 h treatment of HepG2 and Huh7 cells with 10 μM 25-OHC, as compared with the control (**Fig. 2A, B**). We also examined the protein expression of ER stress markers, including ATF4, Chop, phospho-PERK, and phospho-eIF2α by Western blot analysis. All of these markers significantly increased after treatment for 24 h with 10 μM 25-OHC, whereas the expression did not change in cells treated with the vehicle (Fig. 2C, D).

**Fig. 3.**
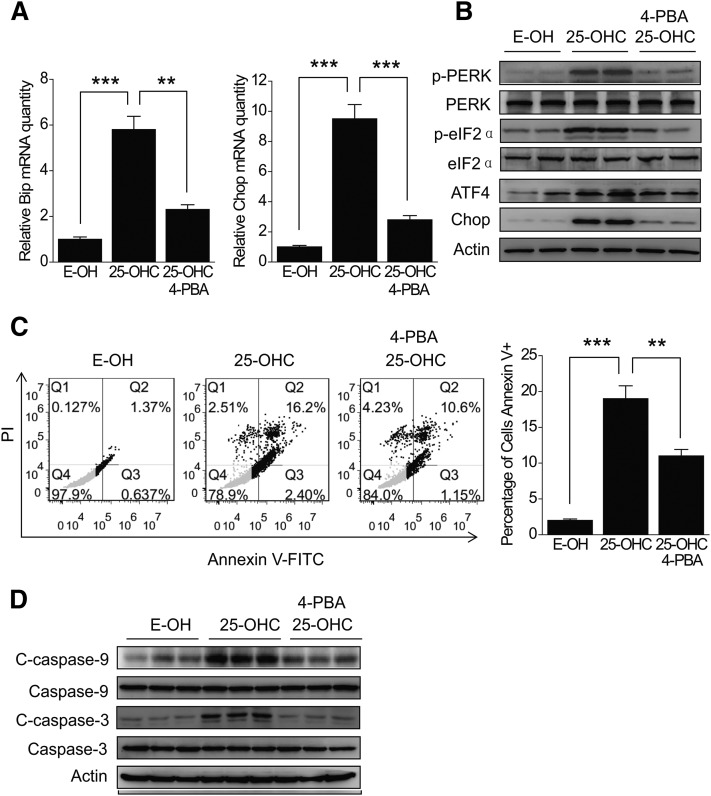
ER stress is involved in 25-OHC-induced apoptosis. A, B: HepG2 cells were treated with or without 2 mM 4-PBA for 1 h, and then incubated with 10 μM 25-OHC for 24 h, relative Bip and Chop mRNA levels were measured by qRT-PCR. ATF4, Chop, phospho-PERK, and phospho-eIF2α protein levels were analyzed by Western blotting. C: HepG2 cells were treated with or without 4-PBA and 25-OHC as indicated above, stained with annexin V-FITC and PI, and analyzed by FCM for cell apoptosis. D: Cleaved caspase-9 and -3 protein levels were analyzed by Western blotting in the 25-OHC-treated HepG2 cells. The data represent mean ± SD from three individual experiments (n = 3, **P < 0.01, ***P < 0.001).

To further confirm the role of 25-OHC in ER stress, HepG2 cells were pretreated with 4-PBA, a chemical molecular chaperone, which has been used to improve the misfolding and mislocalization of proteins and to reduce ER stress-mediated cell damage in vivo and in vitro (30–32). The results showed that, when treated with 10 μM 25-OHC for 24 h, the increase of Bip and Chop mRNAs induced by 25-OHC was significantly reversed (**Fig. 3A**) in the presence of 4-PBA. Expectedly, the ATF4, Chop, phospho-PERK, and phospho-eIF2α protein levels also showed a parallel reduction (Fig. 3B). In order to confirm whether the ER stress induced by 25-OHC contributes to cellular apoptosis, HepG2 cells treated with 25-OHC were subjected to annexin V-FITC/PI double-staining followed by flow cytometry. The results showed an increase in apoptotic cells upon treatment with 25-OHC as compared with the control (Fig. 3C), but the apoptotic cell rate was significantly reduced when the 25-OHC treatment was combined with the addition of 4-PBA (Fig. 3C). We also analyzed the cleaved caspase-9 and -3 levels by Western blotting. Cleavage of the caspases was clearly enhanced in 25-OHC-treated cells, but this response was abolished by addition of 4-PBA, suggesting that apoptosis of the 25-OHC-treated cells is induced by ER stress (Fig. 3D).

### ORP8 is required for apoptosis induced by 25-OHC

**Fig. 4.**
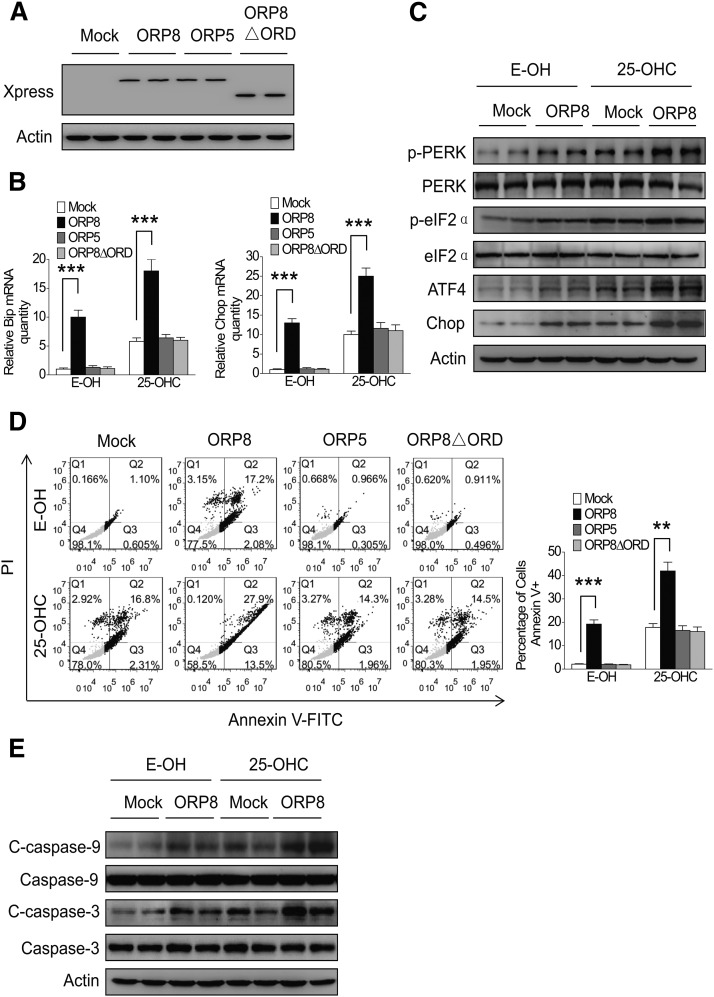
ORP8 overexpression enhances the 25-OHC effect on ER stress and apoptosis. A: HepG2 cells were transfected with ORP8, ORP5, ORP8△ORD cDNA, or empty vector, and the overexpression efficiency was assessed by Western blot analysis. B, C: HepG2 cells were transfected with ORP8, ORP5, and ORP8△ORD cDNA, and then treated with or without 10 μM 25-OHC for 24 h, and relative Bip and Chop mRNA levels were measured by qRT-PCR. ATF4, Chop, phospho-PERK, and phospho-eIF2α protein levels were analyzed by Western blotting. D: HepG2 cells were treated as indicated above, stained with annexin V-FITC and PI, and analyzed by flow cytometry for cell apoptosis. E: Cleaved caspase-9 and -3 protein levels were analyzed by Western blotting. The data represent mean ± SD from three individual experiments (n = 3, **P < 0.01, ***P < 0.001).

We have identified that overexpression of ORP8 induces an ER stress response in HepG2 cells (26), and ORP8 is anchored to ER membranes via its C-terminal transmembrane segment (21). To determine whether ORP8 could mediate 25-OHC-induced ER stress and cell apoptosis, we transfected ORP8 cDNA, ORP8 cDNA lacking the ORD domain (ORP8△ORD), or the related ORP5 cDNA into HepG2 cells and treated them with 25-OHC, followed by analysis of ER stress markers and apoptosis. The results revealed that both 25-OHC treatment and ORP8 overexpression increased the Bip and Chop mRNA expression (**Fig. 4A, B**), while ORP8△ORD and ORP5 failed to do so. Interestingly, the increase of Bip and Chop mRNA induced by 25-OHC was potentiated upon overexpression of ORP8, but not ORP8△ORD or ORP5 (Fig. 4B), and the protein expression of ER stress markers was in conformity with Bip and Chop mRNA levels (Fig. 4C). Analysis of apoptosis by annexin V-FITC/PI staining showed, consistent with the ER stress results, that both 25-OHC treatment and ORP8, but not ORP8△ORD or ORP5 overexpression, induced an increased apoptotic cell rate (Fig. 4D). Furthermore, combined 25-OHC treatment and ORP8, but not ORP8△ORD or ORP5 overexpression, induced a potentiated increase in apoptotic cells (Fig. 4D). Analysis of the cleavage of caspase-9 and -3 by Western blotting further confirmed this observation (Fig. 4E).

**Fig. 5.**
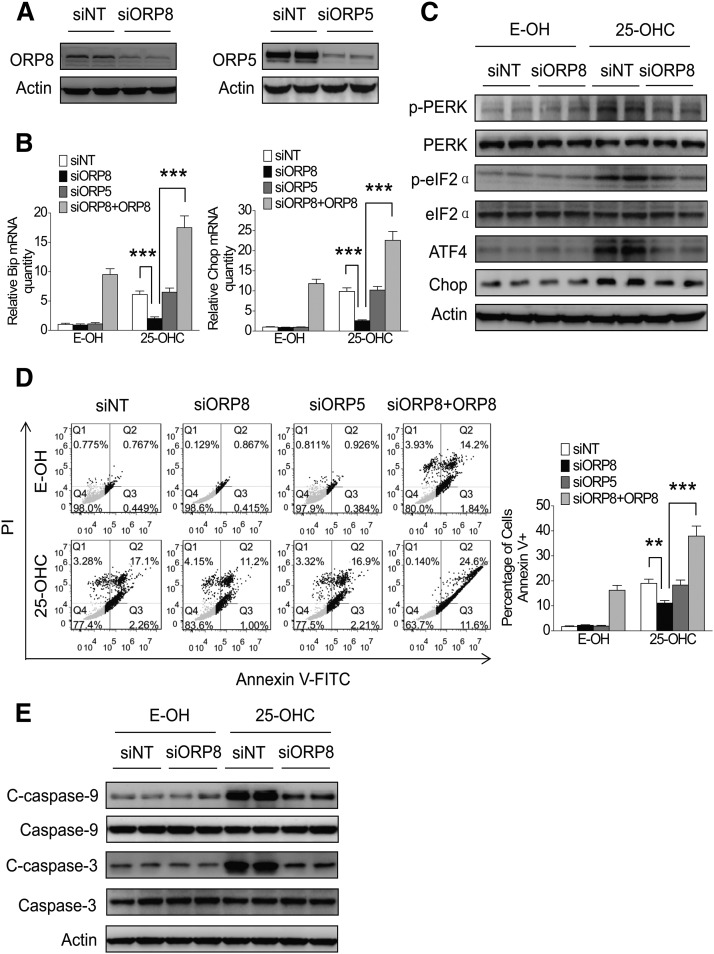
ORP8 knockdown partly abolishes the 25-OHC effect on ER stress and apoptosis. A: HepG2 cells were transfected with siORP8, siORP5, or siNT, and the knockdown efficiency was assessed by Western blot analysis. B, C: HepG2 cells were transfected with siORP8, siORP5, or ORP8 cDNA combined with siORP8, and then treated with or without 10 μM 25-OHC for 24 h, and relative Bip and Chop mRNA levels were measured by qRT-PCR. ATF4, Chop, phospho-PERK, and phospho-eIF2α protein levels were analyzed by Western blotting. D: HepG2 cells were treated as indicated above, stained with annexin V-FITC and PI, and analyzed by flow cytometry for cell apoptosis. E: Cleaved caspase-9 and -3 protein levels were analyzed by Western blotting. The data represent mean ± SD from three individual experiments (n = 3, **P < 0.01, ***P < 0.001).

We next assessed the role of endogenous ORP8 in the effect of 25-OHC-induced ER stress and cell apoptosis by employing a RNA interference approach. ORP8 knockdown (**Fig. 5A**) abolished the increase of Bip and Chop mRNA and ER stress protein markers’ expression induced by 25-OHC, while knockdown of ORP5 failed to do so (Fig. 5B, C). Consistently, annexin V-FITC/PI staining and Western blot analysis of cleaved caspase-9 and -3 showed a reduced degree of apoptosis in HepG2 cells subjected to ORP8 knockdown, but not ORP5 knockdown (Fig. 5D, E). In addition, ORP8 re-overexpression could rescue the decreased Bip and Chop mRNA expression (Fig. 5B) and cell apoptosis (Fig. 5D) upon ORP8 knockdown. These results provide mechanistic evidence supporting the view that ORP8 plays a distinct role in mediating the 25-OHC-induced ER stress and apoptosis in hepatic cells.

## DISCUSSION

The human liver is the central organ for cholesterol homeostasis (33). In extrahepatic tissues, oxysterols are derived from cholesterol through either enzymatic or nonenzymic oxidation (34). Oxysterols exhibit important biological activities in the induction of cell apoptosis, inhibition of cell growth, and regulation of cholesterol metabolism (35, 36). However, excess oxysterols are toxic to cells (37). Therefore, under physiological conditions, excess oxysterols in all extrahepatic tissues are transported to the liver and further metabolized by catabolism into bile acids, esterification to oxysterol esters, sulfation for excretion, and direct efflux from liver cells (34, 38, 39). Oxysterols play a variety of regulatory roles in normal cellular processes, but pathological effects of oxysterol accumulation have also been described, for instance in atherosclerosis, neurologic diseases, and age-onset macular degeneration (40–44). Abnormally high levels of oxysterols act cytotoxic, causing cell death by apoptotic or necrotic processes (7).

HCCs are known to undergo metabolic alterations to sustain faster proliferation (45, 46). Thus, treatments targeting these metabolic alterations may be a new therapeutic strategy (47). HCCs disturb certain cellular functions of the liver and cause pathological alterations in many processes including cholesterol metabolism (48, 49). Previously published data showed that oxysterols induce cytotoxicity and cell death in HepG2 cells (50, 51). Inhibiting ACAT2 leads to the intracellular accumulation of unesterified oxysterols and suppresses the growth of both HCC cell lines and their xenograft tumors (52).

A number of studies have revealed that oxysterols modify cell proliferation capacity and cell fate decisions. In human monocytic THP-1 cells, the oxysterol, 25-OHC, was shown to have dual effects on cell fate: It promoted cell proliferation at concentrations <10 μM, but caused retardation of the THP-1 cell cycle at the G2/M phase and eventually induced apoptosis at >10 μM (5). Similar observations have been made in other cell types (53–55).

In the present study, we found that a low concentration of 7-keto, 7-OHC, 25-OHC, and 27-OHC promoted cell proliferation, while above the critical 10 μM concentration, all of the oxysterols inhibited cell proliferation. Apoptosis is an important biological mechanism that contributes to the maintenance of the integrity of the multicellular organism, and is dependent on the expression of cell-intrinsic suicide machinery. In our study, all of the oxysterols induced HepG2 and Huh7 cell apoptosis in a dose-dependent manner. In order to elucidate the molecular mechanism by which oxysterols induced cell death through apoptosis, we chose 25-OHC, the most extensively studied oxysterol, which has also been described as one of the most toxic species (56, 57). We found that HepG2 cells treated with 10 μM 25-OHC displayed increased ATF4, Chop, phospho-PERK, and phospho-eIF2α protein levels and cell apoptosis. Our results further indicated that 25-OHC induced HepG2 cell apoptosis through ER stress reponse.

Our previous studies have provided evidence for a novel function of ORP8 in the induction of apoptosis in hepatoma cells and suggested that overexpression of ORP8 in HepG2 cells induces an ER stress response, which results in FasL upregulation (26). Now we report that ORP8 may be involved in mediating hepatocellular apoptosis induced by oxysterols. We confirmed that ORP8 overexpression induced apoptosis in HepG2 cells. Importantly, the pro-apoptotic activity of 25-OHC was aggravated by ORP8 overexpression, while ORP8 knockdown could dampen the effect of 25-OHC on apoptosis. These findings suggested that ORP8 is required for the induction of apoptosis by 25-OHC. However, the precise molecular mechanism(s) by which oxysterols act via ORP8 to induce ER stress remains a subject of future study. Incubation with 25-OHC may cause accumulation of 25-OHC fatty acyl esters in the ER due to their defective hydrolysis, thereby activating ER stress signaling (16); ORP8 is anchored in the ER via its C-terminal transmembrane segment and binds 25-OHC (21). One can therefore envision that ORP8 could aggravate the accumulation of 25-OHC esters to enhance the ER stress response. Another possible mechanism by which oxysterols may evoke ER stress and cytotoxicity is distortion of cellular Ca^2+^ homeostasis (58); severe depletion of ER Ca^2+^ stores is known to induce ER stress responses, and distorted ER Ca^2+^ fluxes cause apoptosis via mitochondrial Ca^2+^ overload (59–61). Thus, another possible mechanism through which ORP8 might aggravate the oxysterol effect is interference with the ER calcium fluxes.

In addition to oxysterols, several ORPs have been shown to bind cholesterol and glycerophospholipids. To understand the function of ORP in molecular detail, information on its ligand-binding specificity and affinities for different lipidous ligands is warranted. However, the lack of highly purified and fully functional ORP8 ligand binding domain fusion proteins has thus far hampered detailed biochemical study of its affinities for different sterols. However, the photo-cross-linking by Suchanek et al. (27) suggested that ORP8 also binds cholesterol, and more recent findings suggest that it has the capacity to bind and transport phosphatidylserine and PtdIns-4-P (25).

Our previous work showed that ORP8 expression is downregulated in HCC, which may protect the cancer cells from apoptosis (21). In addition to the role in controlling the apoptosis of hepatic cells, it is possible that the impact of ORP8 on hepatic cholesterol homeostasis could be involved in the carcinogenesis of HCC. Numerous studies have shown increased levels of cholesterol in tumors, including HCC, as compared with normal tissue (62–64). In most cases, the increased cholesterol in cancer cells was caused by the loss of feedback inhibition of cholesterol biosynthesis, uptake of extracellular cholesterol, or efflux of intracellular cholesterol (65–67). We previously reported evidence suggesting that ORP8 is important to maintain the hepatic cholesterol homeostasis by negative control of cholesterol biosynthesis (22). Thus, one can speculate that downregulation of ORP8, a negative regulator of intracellular cholesterol, could be required for an increased level of hepatocellular cholesterol in HCC. This represents another mechanism through which reduced ORP8 expression could benefit the growth of hepatic carcinoma cells.

In conclusion, the present study suggests that ORP8 may mediate 25-OHC-induced ER stress and apoptosis in HepG2 and Huh7 cells. These findings contribute to our understanding of the pathophysiology of HCC cell apoptosis, and may promote the development of new therapies for HCC.

## Supplementary Material

Supplemental Data
