# Supplementary material for: Oxysterol binding protein-related protein 8 mediates the cytotoxicity of 25-hydroxycholesterol
Source: J Lipid Res. 2016 Oct;57(10):1845–53. doi: 10.1194/jlr.M069906 (PMC5036365; doi:10.1194/jlr.M069906)
Supplement: Supplemental Data [file supp_57_10_1845__index.html]

Oxysterol binding protein-related Protein 8 (ORP8) mediates the cytotoxicity of 25-Hydroxycholesterol — Oxysterol binding protein-related protein 8 mediates the cytotoxicity of 25-hydroxycholesterol — Supplemental Data 

# Oxysterol binding protein-related protein 8 mediates the cytotoxicity of 25-hydroxycholesterol

## Supplemental Data

- Supplemental Figure S1 (.pdf, 440 KB) - These additional experiments provide evidence that 10 μM 25-OHC represented an optimal set of treatment parameters for analyses of apoptosis throughout this study .
