# Supplementary material for: Oxysterol binding protein-related protein 8 mediates the cytotoxicity of 25-hydroxycholesterol
Source: J Lipid Res. 2016 Oct;57(10):1845–53. doi: 10.1194/jlr.M069906 (PMC5036365; doi:10.1194/jlr.M069906)
Supplement: Supplemental Data [file 10.1194_M069906_jlr.M069906-1.pdf]

SUPPLEMENTAL INFORMATION:

Oxysterol Binding Protein-Related Protein 8 (ORP8) Mediates the Cytotoxicity of 25-Hydroxycholesterol

Jiwei Li[1,2], Xiuting Zheng[1,2], Ning Lou[3], Wenbin Zhong[1,2], Daoguang Yan[1,2]*

[1]Department of Biotechnology, Jinan University, Guangzhou, 510632, China

[2]the Key Laboratory of Functional Protein Research of Guangdong Higher Education Institutes, Jinan University

[3]State Key Laboratory of Oncology in Southern China, Collaborative Innovation Center of Cancer Medicine, Guangzhou, China.

supplemental Table S1 : The oligonucleotide primers used

Gene	Forward primer 5'- 3'	Reverse primer 5'- 3'
Chop qPCR	GCCTTTCTCCTTTGGGACACTGTCCAGC	CTCGGCGAGTCGCCTCTACTTCCC
Bip qPCR	CCTGGGTGGCGGAACCTTCGATGTG	CTGGACGGGCTTCATAGTAGACCGG
Actin qPCR	GGCATCCTCACCCTGAAGTA	AGGTGTGGTGCCAGATTTTC
ORP8	ATTagatctATGAGTCAGCGCCAAGG	AATgtcgacCTACTTGAACATGAAGTTTATTATG
ORP8△ORD	TCTGAAGAAAACAAAAGCGATACCCGACCATGGGAC	GTCCCATGGTCGGGTATCGCTTTTGTTTTCTTCAGA
ORP5	ATTtctagaATGAAGGAGGAGGCCTTCCT	ATTtctagaCTATTTGAGGATGTGGTTAATGAAC

supplemental Figure S1

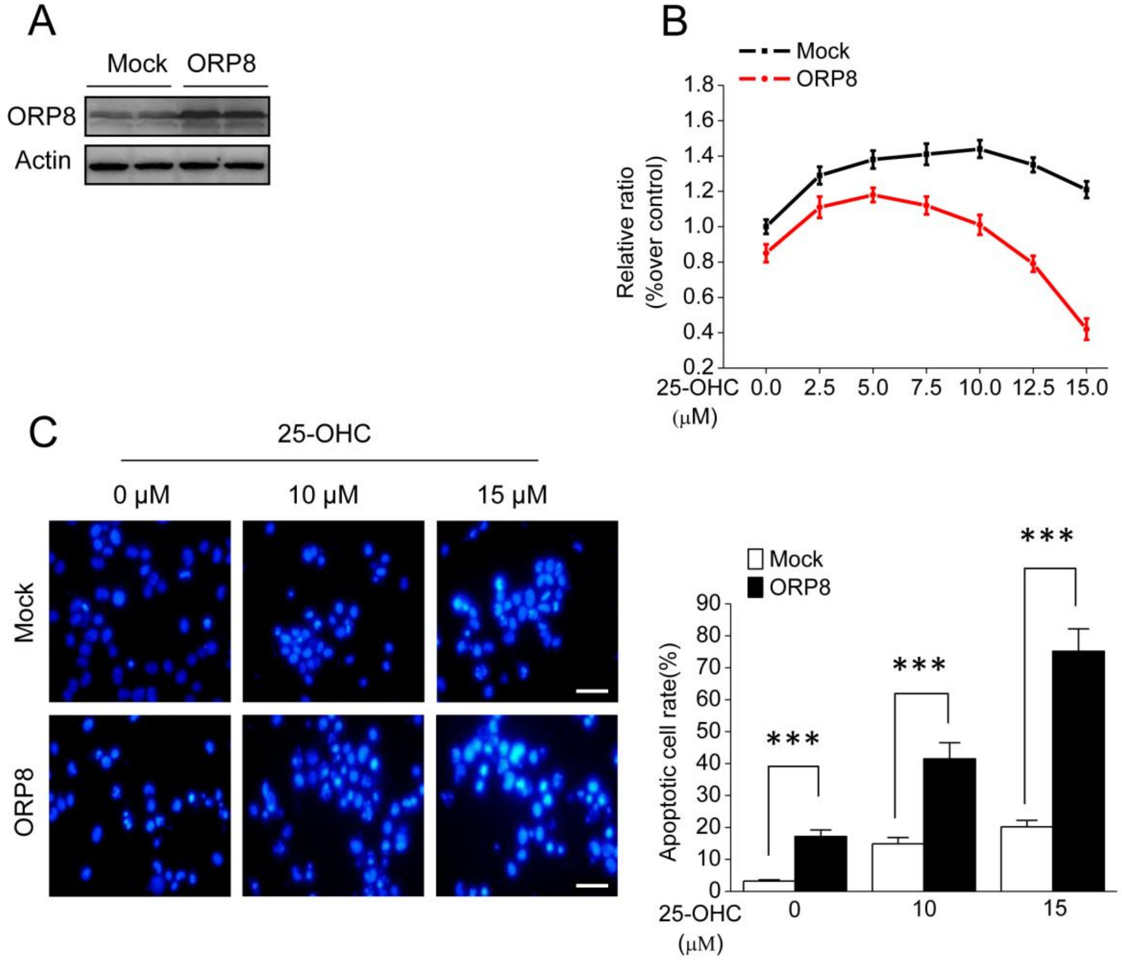

supplemental Figure S1: ORP8 overexpression enhances the 25-OHC effect on cell apoptosis. (A) HepG2 cells were transfected with ORP8 cDNA or empty vector, and the overexpression efficiency was accessed by western blot analysis. (B) HepG2 cells were transfected with ORP8 cDNA or empty vector, then incubated for 24 hr in the presence of different concentrations of 25-OHC, the proliferation rate was detected using CCK-8. (C) HepG2 cells were transfected with ORP8 cDNA or empty vector, then treated with 0, 10, 15 μM 25-OHC

for 24 hr, the nuclear morphology was observed under a microscope after Hoechst 33342 staining. Scale bars, 20 μm. The data represent mean ± S.D. from three individual experiments ($n = 3$, ***$p < 0.001$).
